# MetaWin 3: open-source software for meta-analysis

**DOI:** 10.3389/fbinf.2024.1305969

**Published:** 2024-02-08

**Authors:** Michael S. Rosenberg

**Affiliations:** Center for Biological Data Science, Virginia Commonwealth University, Richmond, VA, United States

**Keywords:** effect sizes, meta-analysis, meta-regression, research synthesis, software

## Abstract

The rise of research synthesis and systematic reviews over the last 25 years has been aided by a series of software packages providing simple and accessible GUI interfaces which are intuitively easy to use by novice analysts and users. Development of many of these packages has been abandoned over time due to a variety of factors, leaving a gap in the software infrastructure available for meta-analysis. To fulfill the continued demand for a GUI-based meta-analytic system, we have now released MetaWin 3 as free, open-source, multi-platform software. MetaWin3 is written in Python and developed from scratch relative to earlier versions. The codebase is available on Github, with pre-compiled executables for both Windows and macOS available from the MetaWin website. MetaWin includes standardized effect size calculations, exploratory and publication bias analyses, and allows for both simple and complex explanatory models of variation within a meta-analytic framework, including meta-regression, using traditional least-squares/moments estimation.

## 1 Introduction

Research synthesis is generally defined as a review of primary research with the intent to integrate findings; meta-analysis is a particular form of quantitative research synthesis with a focus on combining and comparing effect sizes across studies (Glass, 1976; Hedges and Olkin, 1985; Koricheva and Gurevitch, 2013). While meta-analytical studies have been particularly popular in the medical and social sciences, they have been performed for virtually every area of academic research.

The rise of quantitative research synthesis and meta-analysis over the last 25 years was aided by a series of software packages providing simple and accessible GUI interfaces which are intuitively easy to use for novice analysts and users. MetaWin (Rosenberg et al., 1997) was first published as a small, commercial software package that made meta-analytical calculations more accessible to the burgeoning research synthesis community, particularly in the ecological sciences, and helped introduce the use of resampling methods into the meta-analytic statistical repertoire (Adams et al., 1997). Version 2 of the software (Rosenberg et al., 2000) was substantially expanded over the original version, easier to use, and more flexible and powerful. These software have been cited thousands of times in fields including agriculture, anthropology, biology, business, chemistry, economics, education, engineering, forestry, geography, geology, medicine, physics, and psychology, among others.

Versions 1 and 2 of MetaWin were written in Pascal and Delphi and depended on several commercial licensed packages. These developmental environments and decisions combined to restrict the software to the Windows operating system and prevented any practical open-source release as the software was otherwise uncompilable without these components. These also served to restrict further development and updates as the developmental components and systems gradually became outdated and incompatible with newer operating systems.

Despite these limitations, MetaWin has continued to be regularly used and cited for over 20 years after its original release; would-be users still regularly request copies of this relatively ancient software. The long-term popularity has likely been driven by the simple GUI-interface, which contrasts it with powerful, more difficult-to-use alternatives available in R. In an attempt to fill in the gap between MetaWin 2 and the R-based meta-analytic community, OpenMEE (Wallace et al., 2016) was created as open-source, cross-platform software with a GUI interface, but which used R computation on the backend. OpenMEE appeared to fill many of the needs that MetaWin served, but the software became quickly abandoned with development apparently halted in 2016. To fulfill the continued demand for a GUI-based meta-analytic system, we have now released MetaWin 3.

## 2 Materials and methods

MetaWin 3 is free, open-source, and multi-platform, unlike its predecessors. It has been written from scratch relative to the earlier versions, entirely in Python, with the codebase openly available on Github and pre-compiled (using PyInstaller) executables for both Windows and macOS available from the MetaWin website. The code has minimal external dependencies, and relies on only four established, and heavily used Python packages: PyQt6, NumPy, SciPy, and Matplotlib.

The focus of MetaWin is to provide access to meta-analytic fundamentals with an easy-to-use GUI. Output includes citations and references to appropriate literature sources based on method choices made by the users; similarly, all graphical output includes auto-generated sample captions (including references, when appropriate) to ease in interpretation (see below). MetaWin imports and exports data from tables (row × column) in standard text formats (*e.g.*, CSV). Textual output can be exported as plain text, HTML, or markdown. Figures can be exported in a variety of standard graphical formats, including both common vector (*e.g.*, SVG, EPS) and raster (*e.g.*, PNG, TIF, JPG) options.

The major functions in MetaWin are roughly divided into four primary categories: effect size calculations, publication bias exploration, meta-analytic computation, and additional graphical output.

Effect size calculations are for standardized effect measures well established in the meta-analytic literature (Table 1). Effect size calculation is optional within the MetaWin framework, as a user can always import a pre-calculated effect size and its variance for use in the general analytical computation.

**TABLE 1:** Standardized effects size metrics included within MetaWin.

Data type	Effect size
Pairs of means	Hedges’ *d* Hedges and Olkin (1985)
	ln response ratio Hedges et al. (1999)
Two × Two contingency tables	ln odds ratio Mantel and Haenszel (1959); Rosenberg et al. (2000)
	ln relative rate Greenland (1987); L’Abbé et al. (1987); Normand (1999)
	Rate difference DerSimonian and Laird (1986); L’Abbé et al. (1987); Berlin et al. (1989); Normand (1999)
Correlations	Fisher’s Z-transform Fisher (1928)
Probabilities	Logit Mengersen and Gurevitch (2013)

Publication bias exploration (Table 2) includes both graphical approaches (*e.g.,* funnel plots) and analytical methods such as Egger’s Regression, Rank Correlation Tests, and Trim-and-Fill Analysis. Fail-safe number calculation is included as a subcomponent of basic meta-analysis (see below).

**TABLE 2:** Publication bias methods included in MetaWin.

Method
Funnel plots Light and Pillemer (1984)
Pseudo-confidence intervals Sterne and Egger (2001)
Contour confidence intervals Peters et al. (2008)
Power-enhancement (Sunset) plot Kossmeier et al. (2020)
Egger regression Egger et al. (1997)
Rank correlation analysis Begg (1994); Begg and Mazumdar (1994)
Trim and fill analysis Duval and Tweedie (2000b); Duval and Tweedie (2000a)

There are currently 8 primary analytical methods implemented within MetaWin 3, three of which represent simple and exploratory models, while the other five represent more complex explanatory structural models (Table 3), frequently referred to as meta-regression. These include a simple linear model or more complex multivariate models using a general linear model (GLM) regression framework. Most of the methods allow for both fixed- or random-effects variance model implementation; a mixed-effects model meta-analysis (Mengersen et al., 2013) is performed by using the grouped meta-analysis structure with random-effects variance chosen. Heterogeneity is estimated using both *Q*- and *I* ^2^-statistics (Hedges and Olkin, 1985; Higgins and Thompson, 2002; Huedo-Medina et al., 2006). Most analyses include one or more optional resampling tests for determining confidence intervals or significance testing (Adams et al., 1997). All of these methods currently implement traditional moments/least-squares estimators for meta-analysis (Rosenberg, 2013). The basic analysis also includes fail-safe number estimation (Rosenthal, 1979; Orwin, 1983; Rosenberg, 2005).

**TABLE 3:** Primary meta-analytic methods included in MetaWin.

Simple and exploratory analyses	Complex structural analyses
Basic meta-analysis Hedges and Olkin (1985)	Grouped meta-analysis Hedges and Olkin (1985)
Jackknife meta-analysis	Nested group meta-analysis Rosenberg (2013)
Cumulative meta-analysis Chalmers (1991)	Linear meta-regression analysis Hedges and Olkin (1985); Greenland (1987)
	Complex/GLM meta-analysis Hedges and Olkin (1985); Rosenberg et al. (2000)
	Phylogenetic GLM meta-analysis Lajeunesse et al. (2013)

**FIGURE 1:**
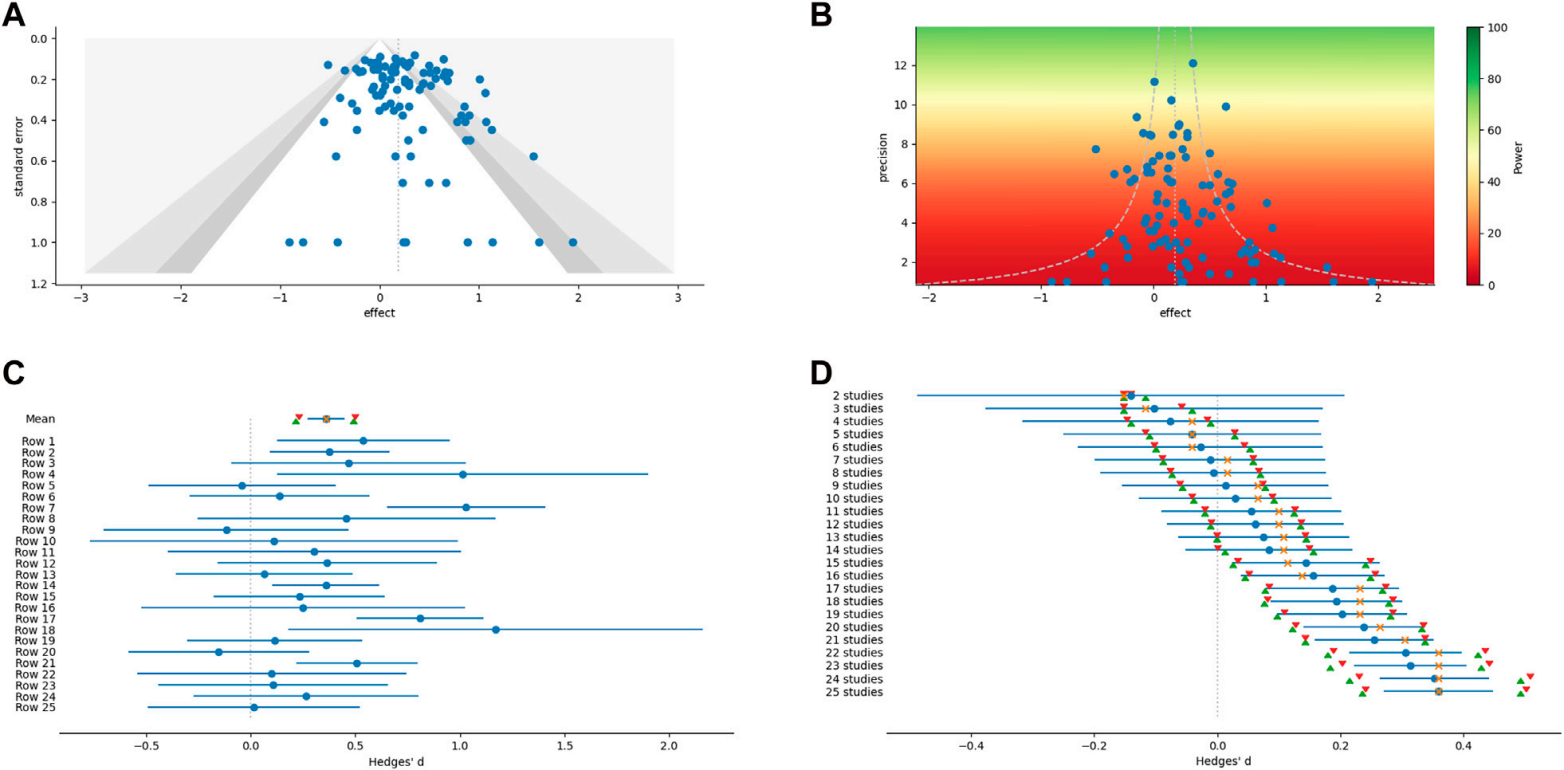
Examples of graphical output from MetaWin. **(A)** Funnel plot with contour confidence intervals. **(B)** A power enhancement funnel plot. **(C)** A forest plot of individual effect sizes and the overall mean and median, including confidence intervals based on both an assumption of a normal distribution and bootstrapping. **(D)** A forest plot of a cumulative meta-analysis.

**FIGURE 2:**
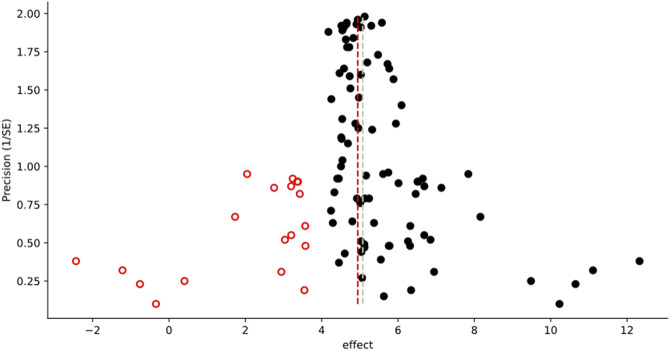
Example of the graphical output from a Trim-and-Fill Analysis. The following caption is automatically generated by MetaWin as part of the figure creation, including the associated reference information: “Funnel plot of effect vs. precision, showing the results of a Trim and Fill Analysis (Duval and Tweedie, 2000a; Duval and Tweedie, 2000b). Original data are represented by black circles, inferred “missing” data by open circles with a fire engine red border. The dashed silver line represents the mean effect size of the original data, the dashed fire engine red line the mean effect size including the inferred data.”

While most of the analytical methods can optionally produce an associated graph or figure, additional figures which have proven useful for meta-analysis can be created outside of the main methods (examples are shown in the panels of Figure 1). These include scatter plots, weighted histograms, forest plots, normal quantile plots (Wang and Bushman, 1998), and Galbraith radial plots (Galbraith, 1988; 1994). Figures are customizable and exportable. Additionally, the underlying data can be exported for re-creation in a user’s preferred plotting software. All figures include automatically generated captions to aid in interpretation and communication (Figure 2).

## 3 Results and discussion

The immediate goal of the current release of MetaWin was to minimally replicate what older versions of the software could do in a modern package, along with adding some obvious enhancements reflecting methodological advancement from the past 20 years. Over half of the analyses listed in Tables 2, 3 were not available in earlier versions of the software. This package now forms a platform to add additional features and analytical methods, for example, potentially including maximum likelihood (Mengersen and Schmid, 2013) and/or Bayesian inference (Schmid and Mengersen, 2013) solutions, which would allow greater flexibility in model specification and distributional assumptions then the currently implemented least-squares approach. Many additional analytical ideas and approaches in meta-analysis have been developed across a broad research synthesis community and could be added to MetaWin in the future, depending on demand, fit, and computational complexity, including additional effect size metrics, analysis models, plots, publication-bias and outlier estimators, etc.

## 4 Availability

MetaWin is free and released under a GPL-3.0 license. Pre-compiled versions of MetaWin for both Windows and macOS can be downloaded directly from https://www.metawinsoft.com. The entire codebase is open-source and available on Github at https://www.github.com/msrosenberg/MetaWin. The help manual can be found on the MetaWin website and is also accessible offline bundled within the pre-compiled executables.

## Data Availability

Publicly available datasets were analyzed in this study. This data can be found here: https://www.github.com/msrosenberg/MetaWin.
